# Leukocyte-Rich Platelet-Rich Plasma’s Clinical Effectiveness in Arthroscopic Rotator Cuff Repair: A Meta-Analysis of Randomized Controlled Trials

**DOI:** 10.3390/bioengineering12060617

**Published:** 2025-06-05

**Authors:** Peiyuan Tang, Meihui Huang, Wenfeng Xiao, Ting Wen, Pavel Volotovsky, Mikhail Gerasimenko, Shiyao Chu, Shuguang Liu, Kai Zhang, Yusheng Li

**Affiliations:** 1 Department of Orthopedics, Xiangya Hospital, Central South University, Changsha 410008, China; tpyzzu@126.com (P.T.); xiaowenfeng@csu.edu.com (W.X.); xyyywenting@163.com (T.W.); 2 National Clinical Research Center for Geriatric Disorders, Xiangya Hospital, Central South University, Changsha 410008, China; 3 Xiangya School of Medicine, Central South University, Changsha 410008, China; 8303221328@csu.edu.cn; 4 Republican Scientific and Practical Center of Traumatology and Orthopedics, 220024 Minsk, Belarus; volotovski@gmail.com (P.V.); gerasimenko@tut.by (M.G.); chushiyao1993@163.com (S.C.); 5 Department of Joint Surgery, Honghui Hospital, Xi’an Jiaotong University, Xi’an 710054, China; orth_shuguang@163.com; 6 Department of Orthopedics, Changde Hospital, Xiangya School of Medicine, Central South University (The First People’s Hospital of Changde City), Changde 415003, China

**Keywords:** rotator cuff, platelet-rich plasma, leukocyte-rich, systematic review, meta-analysis

## Abstract

**Background**: Arthroscopic rotator cuff repair faces high retear risks in multi-tendon injuries due to insufficient biological healing; leukocyte-rich PRP may enhance tendon–bone integration through inflammatory modulation and growth factor release. **Methods**: Four databases including PubMed, Embase, Cochrane Library, and Web of Science were searched until March 2025. Literature screening, quality evaluation, and data extraction were performed according to inclusion and exclusion criteria. GRADE was used to grade the strength of the evidence and the results. **Results**: The main finding of this study was that leukocyte-rich platelet-rich plasma combined with arthroscopic surgery for rotator cuff injuries can improve the Constant Score (MD = 1.13, 95% CI: 0.19, 2.07, *p* = 0.02, I^2^ = 47%), American Shoulder and Elbow Surgeons score (MD = 6.02, 95% CI: 4.67, 7.36, *p* < 0.01, I^2^ = 0%), and University of California, Los Angeles score (MD = 1.20, 95% CI: 0.34, 2.06, *p* < 0.01, I^2^ = 0%) of patients with rotator cuff tear after treatment, and reduce the postoperative Visual Analog Scale score (MD = −0.62, 95% CI: −1.16, −0.08, *p* = 0.02, I^2^ = 83%) of patients. However, there were no statistical differences regarding the Simple Shoulder Test (MD = 0.08, 95% CI: −0.23, 0.39, *p* = 0.61, I^2^ = 5%). **Conclusions**: Based on current evidence, the use of LR-PRP in arthroscopic rotator cuff repair could lessen postoperative pain and improve postoperative functional scores in individuals with rotator cuff injuries.

## 1. Introduction

Rotator cuff injuries are a common shoulder ailment that are often caused by trauma, excessive shoulder movement, or degenerative disease [1]. Shoulder discomfort, decreased strength, and restricted shoulder joint motion are typical signs of a rotator cuff injury [2]. Rotator cuff injuries affect a patient’s ability to perform daily activities, including employment, mobility, and sleep. Regarding the current therapeutic landscape of rotator cuff injuries, arthroscopic surgery is preferred, as it allows patients to quickly recover with few complications [3]. However, the procedure’s reliance on scar-mediated tendon-to-bone healing introduces a critical paradox: although single-tendon repairs achieve satisfactory healing rates, multi-tendon involvement correlates with escalating retear risks due to inadequate biological integration at the fibrovascular interface [4]. In response to this biomechanical–biological divide, platelet-rich plasma (PRP) therapy has emerged as a promising biologic adjunct.

Concentrated platelet solution taken from autologous whole blood is known as PRP [5]. Compared to whole blood, PRP has three to six times the concentration of platelets [6,7]. Clinically, PRP is exclusively prepared from a patient’s own blood (autologous) to avoid immunogenicity. PRP possesses the capability to unleash a diverse array of growth factors [8]. Therefore, PRP has the ability to promote tendon regeneration in cases of injury or degeneration [9]. Leukocyte-rich PRP (LR-PRP), a specialized subset of PRP, retains physiologic or supra-physiologic concentrations of neutrophils, monocytes, and lymphocytes—a critical distinction from standard PRP preparations that exclude leukocytes during centrifugation. While leukocytes in LR-PRP may theoretically impede healing in certain contexts, their role in orchestrating stage-specific inflammatory signaling is critical for tendon–bone interface repair [10]. Growth factors and cytokines can also be released by white blood cells, which are crucial for cell signaling [11]. These biological signals are essential for stimulating cell proliferation, matrix synthesis, and tissue remodeling [12].

The aim of this meta-analysis was to evaluate the clinical efficacy of LR-PRP in rotator cuff repairs by synthesizing data from randomized controlled trials (RCTs). Our hypothesis is that LR-PRP can lessen postoperative pain and enhance postoperative functional scores.

## 2. Materials and Methods

This article has been reported in line with the PRISMA (Preferred Reporting Items for Systematic Reviews and Meta-Analyses) and AMSTAR (Assessing the methodological quality of systematic reviews) guidelines (Supplementary Material S1) [13,14]. In addition, the methodological guidelines of the Cochrane Handbook of Systematic Reviews were followed in the conduct of this systematic review and meta-analysis [15,16]. This systematic review has been registered with PROSPERO (Registration ID: CRD42024498165).

### 2.1. Search Strategy

Four databases were searched: Embase, PubMed, Web of Science, and Cochrane Library, up until March 2025. Two authors searched the databases independently, while a third author helped reach a consensus on any differences. The search process used the mode of combining subject terms and free terms. The main keywords used in the search process were as follows: Rotator Cuff, Teres Minor, Subscapularis, Platelet-rich Plasma, and Infraspinatus. The specific retrieval process and details can be referred to in Supplementary Material S2.

### 2.2. Eligibility Criteria

The eligibility criteria for the systematic review and meta-analysis were based on the PICOS question: P: Patients with rotator cuff tear undergoing arthroscopic repair; I: LR-PRP; C: Standard arthroscopic repair without LR-PRP augmentation (with injection of conventional autologous blood); O: Function score; and S: RCT.

The inclusion criteria were as follows: (1) Partial-thickness injuries classified as Ellman grade II-III involving <50% tendon thickness, or full-thickness injuries ≤3 cm in the sagittal dimension involving 1–2 tendons. (2) More than 150,000 platelets prior to surgery. (3) Preoperative hemoglobin must be at least 11.0 g/dL. (4) The production of PRP adhered to the European Union’s regulatory framework for Substances of Human Origin (SoHO) as outlined in the Directive and its subsequent amendments. (5) The control groups in all included studies underwent standard arthroscopic rotator cuff repair without LR-PRP augmentation. In most trials, the control patients received the conventional autologous blood to maintain blinding. (6) No contagious illnesses.

The exclusion criteria were as follows: (1) Unrepaired tear of subscapular muscle. (2) Patients with osteoarthritis. (3) Prior surgery on the shoulder.

### 2.3. Data Extraction and Quality Assessment

Two authors separately extracted the data and evaluated its quality, while a third author helped to reach a consensus on any differences. Information such as author, number of patients, results, average age, and sex ratio were extracted. The main outcome indicators extracted in this study are as follows: (1) The Constant Score (CS) is a 100-point shoulder-specific evaluation quantifying pain (15 points), activities of daily living (20 points), active range of motion (40 points), and strength (25 points). Scores ≤55 indicate poor function, scores in the range of 56–70 suggest moderate function, scores in the range of 71–85 signify good function, and scores ≥86 reflect excellent outcomes. (2) The Visual Analog Scale (VAS) is a 10 cm continuum (0 = no pain; 10 = worst imaginable pain) assessing pain severity. Clinically significant improvement was defined as a ≥2-point reduction. (3) The Simple Shoulder Test (SST) comprises 12 binary (yes/no) items evaluating basic shoulder functions. Higher affirmative responses (scores in the range of 0–12) reflect better functional capacity. (4) The American Shoulder and Elbow Surgeons score (ASES) is a combination of 50% patient-reported pain (VAS) and 50% physician-assessed function (10 activities). Total scores ≥75 represent satisfactory outcomes. (5) The UCLA Score is a multidimensional 35-point scale grading pain (10), function (10), active forward flexion (5), strength (5), and satisfaction (5). Scores ≤24 denote unsatisfactory results. The Cochrane risk of bias assessment method was used to examine all randomized trials [17].

### 2.4. Grading the Evidence

We graded the strength of the evidence for the outcomes using the Grading of Recommendations Assessment, Development, and Evaluation (GRADE) guidelines [18]. The evidence was rated on the basis of the following five points: risk of bias, inconsistency, indirectness, imprecision, and publication bias. Finally, the strength of the evidence was divided into high, medium, low, and very low.

### 2.5. Statistical Analyses

Review Manager 5.4 was used for all meta-analyses, and a two-sided *p* value below 0.05 was deemed significant. The results of the continuous variables are summarized and represented as a mean difference (MD) with the corresponding 95% confidence interval (CI). The results of the binary variables are summarized and represented as Relative Risk (RR) with the corresponding 95% CI. If considerable heterogeneity was found, the pooled effect sizes were calculated using the random-effect model. If not, a fixed-effect model was used. The amount of heterogeneity among the included studies that could not be entirely attributed to sampling error was assessed using Cochran’s Q statistics and I^2^ statistics. The interpretation of I^2^ values was as follows: low (I^2^: <25%), low to moderate (I^2^: 25–50%), moderate to substantial (I^2^: 50–75%), or substantial (I^2^: >75%). Finally, sensitivity analysis was used to explain the possible heterogeneity of the study.

## 3. Results

### 3.1. Search Results

**Figure 1:**
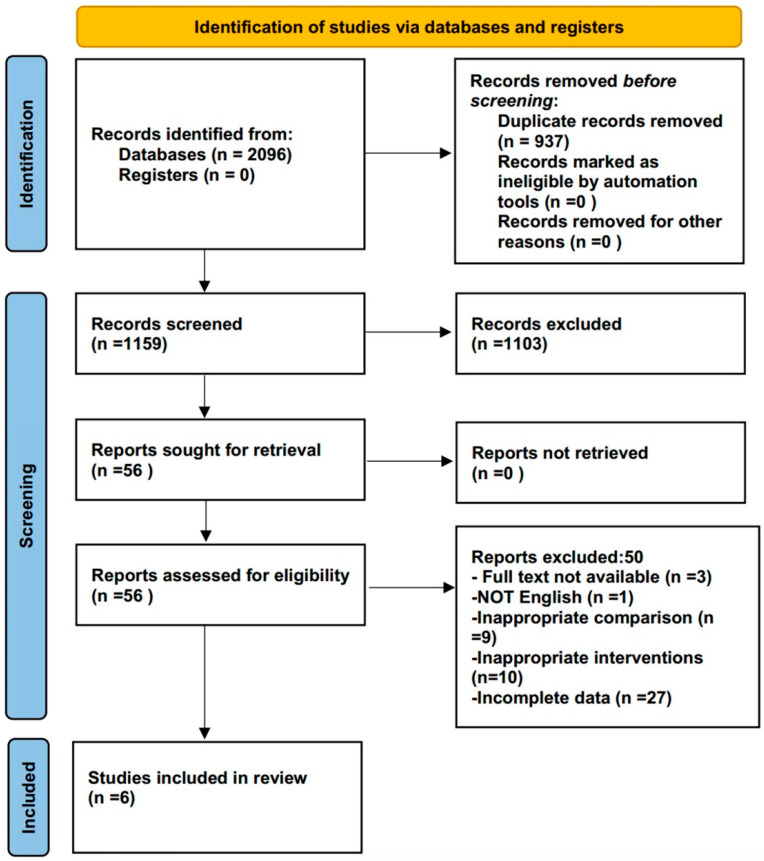
The Preferred Reporting Items for Systematic Reviews and Meta-Analyses (PRISMA) flow diagram is provided to show study selection.

A total of 2096 articles were originally obtained using the search strategy, 937 of which were excluded after removing duplicates, and 1103 were excluded following strict inclusion and exclusion criteria when reading abstracts and titles. Lastly, after reading the full text, 50 more studies were excluded (Supplementary Material S3), resulting in a total of 6 studies that were included. Figure 1 depicts the literature screening procedure.

### 3.2. Study Characteristics

All studies included in this article are RCTs. All studies compared the effects of using and not using LR-PRP after arthroscopic surgery. Five studies lasted for longer than 12 months [19,20,21,22,23]. The average age of the patients in the experimental and control groups was provided in every study. The patients included in this study were mainly older people over 55 years old. The level of evidence for all included studies was II. The proportion of male and female patients included in this paper is basically the same. Table 1 contains the essential features of every study.

### 3.3. Quality Evaluation

**Figure 2:**
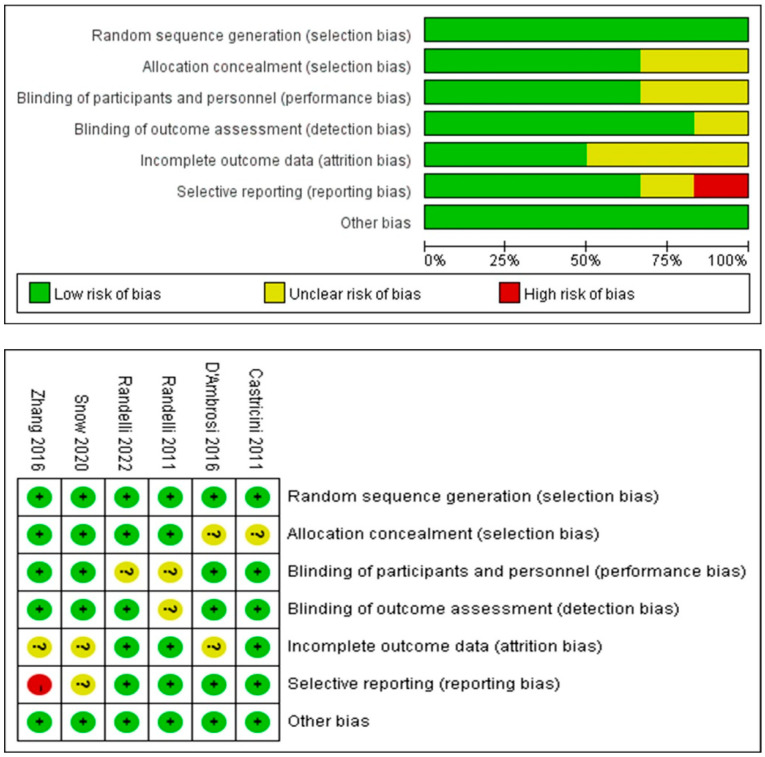
A figure displaying the risk of bias for each of the included randomized studies. The color represents the quality in each of the domains (red = high risk, yellow = uncertain, and green = low risk) [19,20,21,22,23,24].

A total of six RCTs were included, all of which described suitable random sequence generation and randomization methods. However, one study had reporting bias [21]. One study was of medium quality, while the other five were of high quality. (Figure 2).

### 3.4. GRADE Results

**Figure 3:**
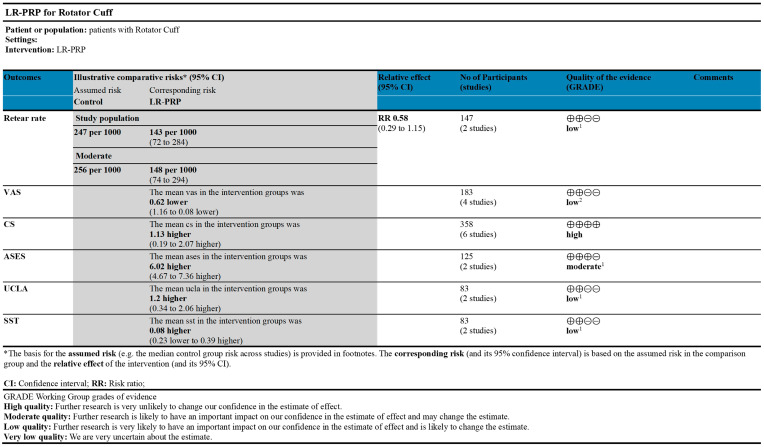
GRADE evidence for outcomes of rotator cuff tears treated with LR-PRP. Platelet-rich plasma (PRP); Leukocyte-rich (LR); Constant Score (CS); Visual Analog Scale (VAS); American Shoulder and Elbow Surgeons score (ASES); University of California, Los Angeles score (UCLA); Simple Shoulder Test (SST). ^1^ I^2^ > 75%; ^2^ The sample size is relatively small.

The GRADE rating results of each outcome indicator are shown in Figure 3. GRADE evidence was classified into three levels: high (CS), medium (ASES), and low (UCLA, VAS, and SST).

### 3.5. Results of Meta-Analysis

#### 3.5.1. Constant Score

**Figure 4:**
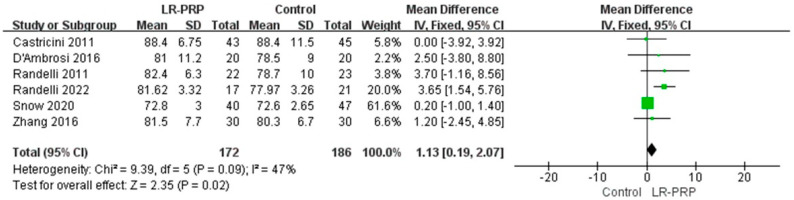
Forest plot of Constant Score outcomes: meta-analysis of LR-PRP vs. control groups using random-effect model [19,20,21,22,23,24].

A total of six RCTs reported a CS [19,20,21,22,23,24]. An analysis using a fixed-effect model showed differences between the LR-PRP and control groups that were statistically significant (MD = 1.13, 95% CI: 0.19, 2.07, *p* = 0.02, I^2^ = 47%) (Figure 4).

#### 3.5.2. Visual Analog Scale

**Figure 5:**
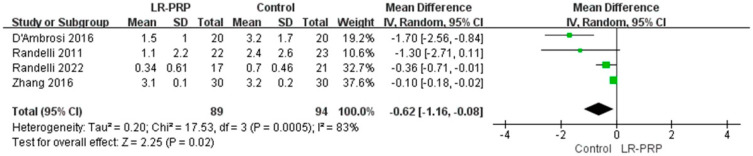
Forest plot of Visual Analog Scale (VAS) pain outcomes: LR-PRP vs. control interventions with high heterogeneity [20,21,23,24].

A total of four RCTs reported VAS [20,21,23,24]. An analysis using a random-effect model showed differences between the LR-PRP and control groups that were statistically significant (MD = −0.62, 95% CI: −1.16, −0.08, *p* = 0.02, I^2^ = 83%) (Figure 5).

#### 3.5.3. Other Results

The meta-analysis results of other outcome indicators are as follows: ASES (MD = 6.02, 95% CI: 4.67, 7.36, *p* < 0.01, I^2^ = 0%), UCLA (MD = 1.20, 95% CI: 0.34, 2.06, *p* < 0.01, I^2^ = 0%), and SST (MD = 0.08, 95% CI: −0.23, 0.39, *p* = 0.61, I^2^ = 5%). Although there was a statistical difference between the ASES score and the UCLA score, when we combined the minimum clinically significant difference, we found that these results had no practical clinical significance. In addition, there was no significant difference in SST score (Supplementary Material S4).

## 4. Discussion

This study’s primary conclusions are that compared with the control group, LR-PRP combined with arthroscopic surgery can improve the CS, ASES, and UCLA after treatment, and reduce the postoperative VAS score of rotator cuff injury patients. However, the experimental and control groups did not show a statistically significantly difference in terms of SST scores. VAS outcome indicators had significant heterogeneity, so we performed a sensitivity analysis on them. After excluding the studies of Zhang [21] and Randelli et al. [23], a fixed-effect model analysis showed statistically significant differences between the LR-PRP and control groups (MD = −1.59, 95% CI: −2.33, −0.85, *p* < 0.01, I^2^ = 0%). The reason for the heterogeneity of the results in Zhang et al. [21] is that the patients received inconsistent doses of LR-PRP. The source of heterogeneity in the results of Randelli et al. [23] is the difference in follow-up time. The results were consistent with our initial hypothesis. After arthroscopic surgery, LR-PRP can reduce postoperative pain or enhance postoperative functional scores in patients with rotator cuff injuries.

This study’s findings were compared with those of previous studies. According to Yinghao Li et al. [25], PRP therapy decreased pain and enhanced functional outcomes. According to Wennan Xu et al. [26], patients’ retear rate and shoulder discomfort were dramatically decreased, and their long-term shoulder function was enhanced following arthroscopic rotator cuff surgery coupled with PRP. Fu-An Yang and colleagues reported that it is advantageous to administer PRP to the bone–tendon interface during surgery [27]. Although the above study did not classify PRP, it still came to conclusions that are similar to ours. However, other researchers have come to conclusions that are the opposite of ours. According to a research study by Pietro Feltri et al. [28], using PRP as an enhancer during arthroscopic surgery for rotator cuff injuries did not significantly improve clinical results. In another study, You-zhi Cai et al. [29] discovered no meaningful statistical difference in clinical outcome scores between treatment with PRP and treatment without PRP in full-layer rotator cuff repair. Jia-Guo Zhao et al. reported similar clinical outcomes in the PRP and control groups [30]. Upon a thorough review of these studies, it was observed that a significant portion of the original research included in these studies concentrated on studying the effects of LP-PRP. These outcomes after surgery in rotator cuff injuries may be a function of LP-PRP rather than LR-PRP.

The meta-analysis revealed substantial heterogeneity in follow-up durations and PRP administration protocols across studies. Specifically, the Randelli P et al. [20] and Randelli PS et al. [23] cohorts represent longitudinal data from the same patient population at 2-year and 10-year intervals. While pooling these datasets introduces temporal heterogeneity, it provides unique insights into the sustained effects of LR-PRP. Nevertheless, the inclusion of overlapping cohorts may inadvertently amplify treatment effect estimates. Additionally, methodological variations in PRP delivery could differentially modulate biologic activity. Snow M et al. [22] administered LR-PRP 10–14 days postoperatively, diverging from intraoperative applications in other trials. This protocol discrepancy may attenuate the regenerative potential of LR-PRP due to delayed inflammatory phase engagement.

Considering the aforementioned studies related to LR-PRP, we suppose that the mechanisms of LR-PRP in patients with rotator cuff injuries is as follows: 1. LR-PRP is rich in growth factors such as platelet-derived growth factor (PDGF), transforming growth factor-beta (TGF-β), vascular endothelial growth factor (VEGF), and epidermal growth factor (EGF) [31]. These factors can promote the proliferation and differentiation of tenocytes and fibroblasts, which are crucial for tendon repair and regeneration. 2. The presence of growth factors like VEGF in LR-PRP could stimulate angiogenesis, which is the formation of new blood vessels [32]. This is critical for supplying nutrients and oxygen to the healing rotator cuff tissues, thereby accelerating the repair process. 3. Growth factors within LR-PRP may also play a role in modulating pain through the inhibition of nociceptive (pain-sensing) pathways, providing symptomatic relief to patients [33]. 4. Factors within LR-PRP can stimulate the synthesis of collagen, an essential component of tendon tissue [34]. Increased collagen synthesis can improve the structural integrity of the repaired tendon.

Another issue is the cost of LR-PRP. When considering the adoption of a medical technology, its value depends on whether it can improve clinical outcomes. The application of LR-PRP in arthroscopic rotator cuff repair has been shown to potentially reduce postoperative discomfort and improve postoperative functional scores, particularly for individuals with rotator cuff injuries. This is highly significant, as it suggests the opportunity for a faster recovery and an improved quality of life for the patients. Therefore, the cost-effectiveness of LR-PRP is not solely about immediate outcomes but is also about long-term patient health. Given that LR-PRP can enhance recovery by lessening postoperative pain and improving postoperative functional scores, it can potentially lead to lower long-term healthcare costs resulting from ongoing concerns or complications. Therefore, the use of LR-PRP in arthroscopic rotator cuff repair can be viewed as a worthwhile expenditure in the pursuit of improved patient outcomes and long-term health benefits.

This study has several limitations. First, only articles written in English were included in this study. Second, there was insufficient data for subgroup analysis. Third, the sample size included in most randomized controlled trials is limited, which reduces the statistical ability to detect clinically significant differences and increases the risk of type II errors. Randomized controlled trials with larger sample sizes are needed in future studies. Fourth, the included studies did not stratify outcomes by clinical risk factors such as diabetes mellitus and smoking status. Finally, the included studies did not address the use of PRP in immunodeficient patients or those with coagulation disorders, which are populations that may require modified biologic strategies. Certain limitations must also be considered when interpreting and drawing conclusions from the data. Therefore, these limitations need to be carefully weighed when interpreting the findings and incorporating them into a comprehensive analysis of the results.

## 5. Conclusions

Based on the current evidence, the use of LR-PRP in arthroscopic rotator cuff repair could lessen postoperative pain and improve postoperative functional scores in individuals with rotator cuff injuries.

## Figures and Tables

**Table 1:** Baseline characteristics of included studies from the literature.

Study	Year	Sample Size		Age		Gender		Tear Size	Follow-Up (M)	Level of Evidence	Outcomes
		LR-PRP	Control	LR-PRP	Control	Man	Woman				
Castricini, R [19]	2011	43	45	55.5 ± 7.8	55.2 ± 8.0	40	48	Small to Moderate	20	II	CS
Randelli, P [20]	2011	22	23	61.6 ± 8.3	59.5 ± 10.7	21	24	Small to Moderate	24	II	VAS, CS, UCLA, SST
Zhang Z [21]	2016	30	30	57.2 ± 7.4	56.9 ± 6.0	31	29	Small (<1 cm)	12	II	VAS, CS
D’Ambrosi, R [24]	2016	20	20	57.9 ± 8.7	62.0 ± 10.0	19	21	Small to Moderate	6	II	VAS, CS
Snow M [22]	2020	40	47	59.8 ± 7.37	62.6 ± 7.37	46	41	Moderate (1–3 cm)	12	II	CS, ASES
Randelli PS [23]	2022	17	21	61.5 ± 3.3	61.56 ± 8.24	23	15	Small to Moderate	120	II	VAS, CS, UCLA, ASES, SST

Abbreviations: Not access (NA); Platelet-rich plasma (PRP); Leukocyte-rich (LR); Constant Score (CS); Visual Analog Scale (VAS); American Shoulder and Elbow Surgeons score (ASES); University of California, Los Angeles score (UCLA); Simple Shoulder Test (SST).

## Data Availability

Data are contained within the article.

## Supplementary Materials

The following supporting information can be downloaded at https://www.mdpi.com/article/10.3390/bioengineering12060617/s1, Supplementary Material S1: PRISMA 2020 Checklist; Supplementary Material S2: Detailed search strategies for each database; Supplementary Material S3: Excluded studies and their reasons after reading the full text; Supplementary Material S4: Additional results of the meta-analysis.

## Abbreviations

The following abbreviations are used in this manuscript:

PRP	Platelet-rich plasma
LR	Leukocyte-rich
CS	Constant Score
VAS	Visual Analog Scale
ASES	American Shoulder and Elbow Surgeons score
UCLA	University of California, Los Angeles score
SST	Simple Shoulder Test
