# Supplementary material for: Leukocyte-Rich Platelet-Rich Plasma’s Clinical Effectiveness in Arthroscopic Rotator Cuff Repair: A Meta-Analysis of Randomized Controlled Trials
Source: Bioengineering (Basel). 2025 Jun 5;12(6):617. doi: 10.3390/bioengineering12060617 (PMC12189123; doi:10.3390/bioengineering12060617)
Supplement: Supplementary file 1 [file bioengineering-12-00617-s001.zip › Supplementary material 2.pdf]

Supplementary material 2: Detailed search strategies for each database.

Pubmed：

Search: ((Rotator Cuff[MeSH Terms]) OR (((((((Rotator Cuff[Title/Abstract]) OR (Cuff, Rotator[Title/Abstract])) OR (Rotator Cuffs[Title/Abstract])) OR (Teres Minor[Title/Abstract])) OR (Subscapularis[Title/Abstract])) OR (Infraspinatus[Title/Abstract])) OR (Supraspinatus[Title/Abstract]))) AND ((Platelet-rich Plasma[MeSH Terms]) OR ((((Platelet-rich Plasma[Title/Abstract]) OR (Plasma, Platelet-Rich[Title/Abstract])) OR (Platelet Rich Plasma[Title/Abstract])) OR (Platelet-rich Plasma[Title/Abstract])))

Web of science：

1: (((TS=(Platelet-rich Plasma)) OR TS=(Plasma, Platelet-Rich)) OR TS=(Platelet Rich Plasma)) OR TS=(Platelet-rich Plasma)

2: ((((((TS=(Rotator Cuff)) OR TS=(Cuff, Rotator)) OR TS=(Rotator Cuffs)) OR TS=(Teres Minor)) OR TS=(Subscapularis)) OR TS=(Infraspinatus)) OR TS=(Supraspinatus)

3: #2 AND #1

Embase：

#7. #5 AND #6

#6. #3 OR #4

#5. #1 OR #2

#4. 'rotator cuff':ab,ti OR 'cuff, rotator':ab,ti OR
'rotator cuffs':ab,ti OR 'teres minor':ab,ti OR
subscapularis:ab,ti OR infraspinatus:ab,ti OR
supraspinatus:ab,ti

#3. 'rotator cuff injury'/exp

#2. 'plasma, platelet-rich':ab,ti OR 'platelet rich
plasma':ab,ti OR 'platelet-rich plasma':ab,ti

#1. 'thrombocyte rich plasma'/exp OR 'thrombocyte
rich plasma'

Cochrane:

#1 MeSH descriptor: [Platelet-Rich Plasma] explode all trees

#2 (Platelet-rich Plasma):ti,ab,kw OR (Plasma, Platelet-Rich):ti,ab,kw OR (Platelet Rich Plasma):ti,ab,kw OR (Platelet-rich Plasma):ti,ab,kw

#3 MeSH descriptor: [Rotator Cuff] explode all trees

#4 (Rotator Cuff):ti,ab,kw OR (Cuff, Rotator):ti,ab,kw OR (Rotator Cuffs):ti,ab,kw OR (Teres Minor):ti,ab,kw OR (Subscapularis):ti,ab,kw

#5 (Infraspinatus):ti,ab,kw OR (Supraspinatus):ti,ab,kw

#6 #1 OR #2

#7 #3 OR #4 OR #5

#8 #6 AND #7
