# Supplementary material for: Leukocyte-Rich Platelet-Rich Plasma’s Clinical Effectiveness in Arthroscopic Rotator Cuff Repair: A Meta-Analysis of Randomized Controlled Trials
Source: Bioengineering (Basel). 2025 Jun 5;12(6):617. doi: 10.3390/bioengineering12060617 (PMC12189123; doi:10.3390/bioengineering12060617)
Supplement: Supplementary file 1 [file bioengineering-12-00617-s001.zip › Supplementary material 3.pdf]

Supplementary material 3: Excluded studies and their reasons after reading the full text.

Excluded studies	Reason
[8]	Inappropriate interventions
[30]	Inappropriate interventions
[41]	Inappropriate interventions
[43]	Inappropriate interventions
[1]	Inappropriate interventions
[11]	Inappropriate interventions
[37]	Inappropriate interventions
[50]	Inappropriate interventions
[9]	Inappropriate interventions
[14]	Inappropriate interventions
[44]	NOT English
[7]	Inappropriate comparison
[12]	Inappropriate comparison
[29]	Inappropriate comparison
[49]	Inappropriate comparison
[42]	Inappropriate comparison
[31]	Inappropriate comparison
[34]	Inappropriate comparison
[39]	Inappropriate comparison
[48]	Inappropriate comparison
[2]	Full text not available
[10]	Full text not available
[36]	Full text not available
[3]	Incomplete data
[5]	Incomplete data
[6]	Incomplete data
[13]	Incomplete data
[15]	Incomplete data
[18]	Incomplete data
[19]	Incomplete data
[20]	Incomplete data
[23]	Incomplete data
[24]	Incomplete data
[25]	Incomplete data
[26]	Incomplete data
[27]	Incomplete data
[28]	Incomplete data

32	**Incomplete data**
35	**Incomplete data**
38	**Incomplete data**
45	**Incomplete data**
46	**Incomplete data**
47	**Incomplete data**
4	**Incomplete data**
16	**Incomplete data**
17	**Incomplete data**
21	**Incomplete data**
22	**Incomplete data**
33	**Incomplete data**
40	**Incomplete data**

References:

1. Aurégan JC, Klouche S, Levy B, Bauer T, Rousselin B, Ferrand M, et al. Autologous Conditioned Plasma for tendon healing following arthroscopic rotator cuff repair. Prospective comparative assessment with magnetic resonance arthrography at 6 months' follow-up. Orthopaedics and Traumatology: Surgery and Research 2019;105(2):245-9. doi:10.1016/j.otsr.2019.01.003
2. Cai YU, Sun Z, Liao B, Song Z, Xiao T, Zhu P. Sodium Hyaluronate and Platelet-Rich Plasma for Partial-Thickness Rotator Cuff Tears. Med Sci Sports Exerc 2019;51(2):227-33. doi:10.1249/mss.0000000000001781
3. de Dios Berna-Mestre J, Fernandez C, Carbonell G, Garcia A, Antonio Garcia-Vidal J, Medina i Mirapeix F, et al. Influence of Acromial Morphologic Characteristics and Acromioclavicular Arthrosis on the Effect of Platelet-Rich Plasma on Partial Tears of the Supraspinatus Tendon. American Journal of Roentgenology 2020;215(4):954-62. doi:10.2214/ajr.19.22331
4. Di Benedetto P, Di Benedetto ED, Beltrame A, Gisonni R, Cainero V, Causero A. Arthroscopic rotator cuff repair with or without PrP: our experience. Acta Biomed 2016;87 Suppl 1:75-83.
5. El Gharbawy NH, Labib HS. Role of Platelet Rich Plasma (PRP) injection in treatment of rotator cuff tear. Egyptian Rheumatology and Rehabilitation 2020;47(1). doi:10.1186/s43166-020-00032-3
6. Ellen MI, Lin C. Common Injuries of the Weekend Athlete. Medical Clinics of North America 2020;104(2):313-+. doi:10.1016/j.mcna.2019.10.010
7. Freitag J, Shah K, Wickham J, Tenen A. Effect of autologous adipose-derived mesenchymal stem cell therapy in combination with autologous platelet-rich plasma in the treatment of elbow tendinopathy. Bmj Case Reports 2020;13(6). doi:10.1136/bcr-2020-234592
8. Grad R, Ebell MH. Top POEMs of 2019 Consistent with the Principles of the Choosing Wisely Campaign. American Family Physician 2020;102(11):673-8.
9. Gumina S, Campagna V, Ferrazza G, Giannicola G, Fratalocchi F, Milani A, et al. Use of

platelet-leukocyte membrane in arthroscopic repair of large rotator cuff tears: a prospective randomized study. The Journal of Bone and Joint Surgery American Volume 2012;94(15):1345-52. doi:10.2106/JBJS.K.00394

10. Gwinner C, Scheibel M. Editorial Commentary: Platelet-Rich Plasma in the Treatment of Rotator Cuff Tears-From Hero to Zero? Arthroscopy 2020;36(3):658-9. doi:10.1016/j.arthro.2019.11.105

11. Hitchen J, Wragg NM, Shariatzadeh M, Wilson SL. Platelet Rich Plasma as a Treatment Method for Rotator Cuff Tears. SN Comprehensive Clinical Medicine 2020;2(11):2293-9. doi:10.1007/s42399-020-00500-z

12. Hurd JL, Facile TR, Weiss J, Hayes M, Hayes M, Furia JP, et al. Safety and efficacy of treating symptomatic, partial-thickness rotator cuff tears with fresh, uncultured, unmodified, autologous adipose-derived regenerative cells (UA-ADRCs) isolated at the point of care: a prospective, randomized, controlled first-in-human pilot study. Journal of Orthopaedic Surgery and Research 2020;15(1). doi:10.1186/s13018-020-01631-8

13. Kataoka T, Mifune Y, Inui A, Nishimoto H, Kurosawa T, Yamaura K, et al. Combined therapy of platelet-rich plasma and basic fibroblast growth factor using gelatin-hydrogel sheet for rotator cuff healing in rat models. Journal of Orthopaedic Surgery and Research 2021;16(1). doi:10.1186/s13018-021-02771-1

14. Kirschner JS, Cheng J, Hurwitz N, Santiago K, Lin E, Beatty N, et al. Ultrasound-guided percutaneous needle tenotomy (PNT) alone versus PNT plus platelet-rich plasma injection for the treatment of chronic tendinosis: A randomized controlled trial. Pm&R 2021;13(12):1340-9. doi:10.1002/pmrj.12583

15. Koscso JM, McElheny K, Carr JB, II, Hippensteel KJ. Lower Extremity Muscle Injuries in the Overhead Athlete. Current Reviews in Musculoskeletal Medicine 2022;15(6):500-12. doi:10.1007/s12178-022-09786-z

16. Kumar A, Singh H, Rehncy JS, Sandhu KS, Sahni G. Platelet-rich plasma versus steroid injection in rotator cuff tendinopathies – A comparative study. National Journal of Physiology, Pharmacy and Pharmacology 2022;12(11):1933-8. doi:10.5455/njppp.2022.12.03143202200020420222

17. Kumar SP, Venkateshulu E, Kartik K, AquibShakeel M. The role of platelet rich plasma in patients with rotator cuff tendinopathy. European Journal of Molecular and Clinical Medicine 2022;9(6):1772-9.

18. Kwong CA, Woodmass JM, Gusnowski EM, Bois AJ, Leblanc J, More KD, et al. Platelet-Rich Plasma in Patients With Partial-Thickness Rotator Cuff Tears or Tendinopathy Leads to Significantly Improved Short-Term Pain Relief and Function Compared With Corticosteroid Injection: A Double-Blind Randomized Controlled Trial. Arthroscopy-the Journal of Arthroscopic and Related Surgery 2021;37(2):510-7. doi:10.1016/j.arthro.2020.10.037

19. Lacheta L, Braun S. Limited evidence for biological treatment measures for cartilage and tendon injuries of the shoulder. Knee Surgery Sports Traumatology Arthroscopy 2022;30(4):1132-7. doi:10.1007/s00167-021-06499-7

20. Lafrance S, Charron M, Roy J-S, Dyer J-O, Fremont P, Dionne CE, et al. Diagnosing, Managing, and Supporting Return to Work of Adults With Rotator Cuff Disorders: A Clinical Practice Guideline. Journal of Orthopaedic & Sports Physical Therapy 2022;52(10):647-+. doi:10.2519/jospt.2022.11306

21. Lana JFSD, da Fonseca LF, Macedo RDR, Mosaner T, Murrell W, Kumar A, et al. Platelet-rich plasma vs bone marrow aspirate concentrate: An overview of mechanisms of action and orthobiologic synergistic effects. World Journal of Stem Cells 2021;13(2):155-67. doi:10.4252/wjsc.v13.i2.155
22. Lavoie-Gagne O, Farah G, Lu Y, Mehta N, Parvaresh KC, Forsythe B. Physical Therapy Combined With Subacromial Cortisone Injection Is a First-Line Treatment Whereas Acromioplasty With Physical Therapy Is Best if Nonoperative Interventions Fail for the Management of Subacromial Impingement: A Systematic Review and Network Meta-Analysis. Arthroscopy - Journal of Arthroscopic and Related Surgery 2022;38(8):2511-24. doi:10.1016/j.arthro.2022.02.008
23. Lee MJ, Yoon KS, Oh S, Shin S, Jo CH. Allogenic Pure Platelet-Rich Plasma Therapy for Adhesive Capsulitis A Bed-to-Bench Study With Propensity Score Matching Using a Corticosteroid Control Group. American Journal of Sports Medicine 2021;49(9):2309-20. doi:10.1177/03635465211018636
24. Lei L, Zhang C, Sun F-H, Xie Y, Liang B, Wang L, et al. Research Trends on the Rotator Cuff Tendon: A Bibliometric Analysis of the Past 2 Decades. Orthopaedic Journal of Sports Medicine 2021;9(1). doi:10.1177/2325967120973688
25. Li M, Wang K, Zhang H, Fang C, Liu H, Zhang Y. Clinical Evaluations of Intraoperative Injection of Platelet-Rich Plasma in Arthroscopic Single-Row Rotator Cuff Repair at 2-Year Follow-Up. Biomed Research International 2021;2021. doi:10.1155/2021/6675097
26. Lin K-Y, Chen P, Chen AC-Y, Chan Y-S, Lei KF, Chiu C-H. Leukocyte-Rich Platelet-Rich Plasma Has Better Stimulating Effects on Tenocyte Proliferation Compared With Leukocyte-Poor Platelet-Rich Plasma. Orthopaedic Journal of Sports Medicine 2022;10(3). doi:10.1177/23259671221084706
27. Liu B, Jeong HJ, Yeo JH, Oh JH. Efficacy of Intraoperative Platelet-Rich Plasma Augmentation and Postoperative Platelet-Rich Plasma Booster Injection for Rotator Cuff Healing: A Randomized Controlled Clinical Trial. Orthopaedic Journal of Sports Medicine 2021;9(6). doi:10.1177/23259671211006100
28. Lui PPY, Zhang X, Yao S, Sun H, Huang C. Roles of Oxidative Stress in Acute Tendon Injury and Degenerative Tendinopathy-A Target for Intervention. International Journal of Molecular Sciences 2022;23(7). doi:10.3390/ijms23073571
29. Maillot C, Martellotto A, Demezon H, Harly E, Le Huec J-C. Multiple Treatment Comparisons for Large and Massive Rotator Cuff Tears: A Network Meta-analysis. Clinical Journal of Sport Medicine 2021;31(6):501-8. doi:10.1097/jsm.0000000000000786
30. Montgomery SR, Petrigliano FA, Gamradt SC. Biologic augmentation of rotator cuff repair. Current reviews in musculoskeletal medicine 2011;4(4):221-30. doi:10.1007/s12178-011-9095-6
31. Nguyen RT, Borg-Stein J, McInnis K. Applications of Platelet-Rich Plasma in Musculoskeletal and Sports Medicine: An Evidence-Based Approach. PM and R 2011;3(3):226-50. doi:10.1016/j.pmrj.2010.11.007
32. Nixon AJ, Watts AE, Schnabel LV. Cell- and gene-based approaches to tendon regeneration. Journal of Shoulder and Elbow Surgery 2012;21(2):278-94. doi:10.1016/j.jse.2011.11.015
33. O’Dowd A. Update on the Use of Platelet-Rich Plasma Injections in the Management of Musculoskeletal Injuries: A Systematic Review of Studies From 2014 to 2021. Orthopaedic

Journal of Sports Medicine 2022;10(12). doi:10.1177/23259671221140888
34. Peng Y, Guanglan W, Jia S, Zheng C. Leukocyte-rich and Leukocyte-poor Platelet-rich Plasma in Rotator Cuff Repair: A Meta-analysis. International journal of sports medicine 2022;43(11):921-30. doi:10.1055/a-1790-7982
35. Proctor CS. Rotator cuff repair augmented with endogenous fibrin clot. Arthroscopy techniques 2012;1(1):e79-82. doi:10.1016/j.eats.2012.03.002
36. Rohman ML, Snow M. Use of biologics in rotator cuff disorders: Current concept review. J Clin Orthop Trauma 2021;19:81-8. doi:10.1016/j.jcot.2021.05.005
37. Sakabe T, Sakai T. Musculoskeletal diseasestendon. British Medical Bulletin 2011;99(1):211-25. doi:10.1093/bmb/ldr025
38. Sheth U, Simunovic N, Klein G, Fu F, Einhorn TA, Schemitsch E, et al. Efficacy of Autologous Platelet-Rich Plasma Use for Orthopaedic Indications: A Meta-Analysis. Journal of Bone and Joint Surgery-American Volume 2012;94A(4):298-307. doi:10.2106/jbjs.K.00154
39. Solomon DJ. Editorial Commentary: Leukocyte-Poor Platelet-Rich Plasma Decreases Retear Rate in Arthroscopic Rotator Cuff Repair: Platelet-Rich Plasma Type Matters. Arthroscopy - Journal of Arthroscopic and Related Surgery 2021;37(8):2625-6. doi:10.1016/j.arthro.2021.04.028
40. Steinert AF, Middleton KK, Araujo PH, Fu FH. Platelet-Rich Plasma in Orthopaedic Surgery and Sports Medicine: Pearls, Pitfalls, and New Trends in Research. Operative Techniques in Orthopaedics 2012;22(2):91-103. doi:10.1053/j.oto.2011.10.004
41. Textor JA, Tablin F. Activation of Equine Platelet-Rich Plasma: Comparison of Methods and Characterization of Equine Autologous Thrombin. Veterinary Surgery 2012;41(7):784-94. doi:10.1111/j.1532-950X.2012.01016.x
42. Van Schaik KD, Lee KS. Orthobiologics: Diagnosis and Treatment of Common Tendinopathies. Semin Musculoskelet Radiol 2021;25(6):735-44. doi:10.1055/s-0041-1735475
43. Wei L-C, Gao S-G, Xu M, Jiang W, Tian J, Lei G-H. A novel hypothesis: The application of platelet-rich plasma can promote the clinical healing of white-white meniscal tears. Medical Science Monitor 2012;18(8):HY47-HY50. doi:10.12659/msm.883254
44. Wimmer MD, Vavken P, Nehrer S, Valderrabano V. Evidence in sports orthopedics: Application options and evidence for PRP (platelet-rich plasma) in orthopedics. Sport-Orthopadie - Sport-Traumatologie 2012;28(4):244-9. doi:10.1016/j.orthtr.2012.10.001
45. Yilmaz I, Akkaya S, Isyar M, Batmaz AG, Guler O, Oznam K, et al. Is there a treatment protocol in which platelet-rich plasma is effective? Journal of orthopaedics 2016;13(4):316-21. doi:10.1016/j.jor.2016.06.027
46. Zhang JY, Fabricant PD, Ishmael CR, Wang JC, Petrigliano FA, Jones KJ. Utilization of Platelet-Rich Plasma for Musculoskeletal Injuries An Analysis of Current Treatment Trends in the United States. Orthopaedic Journal of Sports Medicine 2016;4(12). doi:10.1177/2325967116676241
47. Zhang L, Chen S, Chang P, Bao N, Yang C, Ti Y, et al. Harmful Effects of Leukocyte-Rich Platelet-Rich Plasma on Rabbit Tendon Stem Cells In Vitro. American Journal of Sports Medicine 2016;44(8):1941-51. doi:10.1177/0363546516644718
48. Zumstein MA, Berger S, Schober M, Boileau P, Nyffeler RW, Horn M, et al. Leukocyte- and platelet-rich fibrin (L-PRF) for long-term delivery of growth factor in rotator cuff repair: Review, preliminary results and future directions. Current Pharmaceutical Biotechnology

2012;13(7):1196-206. doi:10.2174/138920112800624337

49. Zumstein MA, Bielecki T, Ehrenfest DMD. The Future of Platelet Concentrates in Sports Medicine: Platelet-Rich Plasma, Platelet-Rich Fibrin, and the Impact of Scaffolds and Cells on the Long-term Delivery of Growth Factors. Operative Techniques in Sports Medicine 2011;19(3):190-7. doi:10.1053/j.otsm.2011.01.001

50. Zumstein MA, Rumian A, Thélu CÉ, Lesbats V, O'Shea K, Schaer M, et al. SECEC Research Grant 2008 II: Use of platelet- and leucocyte-rich fibrin (L-PRF) does not affect late rotator cuff tendon healing: a prospective randomized controlled study. Journal of Shoulder and Elbow Surgery 2016;25(1). doi:10.1016/j.jse.2015.09.018
