# Supplementary material for: Leukocyte-Rich Platelet-Rich Plasma’s Clinical Effectiveness in Arthroscopic Rotator Cuff Repair: A Meta-Analysis of Randomized Controlled Trials
Source: Bioengineering (Basel). 2025 Jun 5;12(6):617. doi: 10.3390/bioengineering12060617 (PMC12189123; doi:10.3390/bioengineering12060617)
Supplement: Supplementary file 1 [file bioengineering-12-00617-s001.zip › Supplementary material 4.pdf]

Supplementary material 4: Additional results of the meta-analysis.

Study or Subgroup	LR-PRP Mean	LR-PRP SD	LR-PRP Total	Control Mean	Control SD	Control Total	Weight	Mean Difference IV, Fixed, 95% CI
Randelli 2022	100	1.46	17	93.33	7.9	21	15.3%	6.67 [3.22, 10.12]
Snow 2020	80.1	3.33	40	74.2	3.63	47	84.7%	5.90 [4.44, 7.36]
**Total (95% CI)**			**57**			**68**	**100.0%**	**6.02 [4.67, 7.36]**

Heterogeneity: Chi² = 0.16, df = 1 (P = 0.69); I² = 0%
Test for overall effect: Z = 8.75 (P < 0.00001)

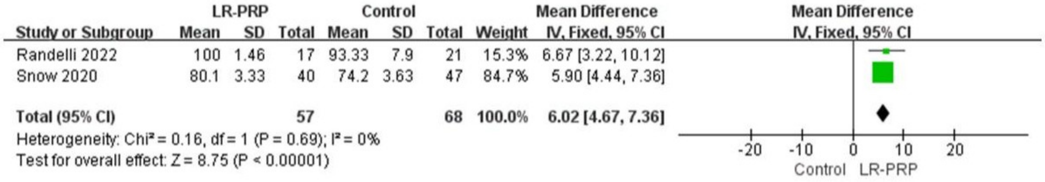

Supplementary Figure 1. Forest plots of American Shoulder and Elbow Surgeons score.

Study or Subgroup	LR-PRP Mean	LR-PRP SD	LR-PRP Total	Control Mean	Control SD	Control Total	Weight	Mean Difference IV, Fixed, 95% CI
Randelli 2011	11.3	0.9	22	10.9	1.4	23	20.0%	0.40 [-0.28, 1.08]
Randelli 2022	12	0.25	17	12	0.75	21	80.0%	0.00 [-0.34, 0.34]
**Total (95% CI)**			**39**			**44**	**100.0%**	**0.08 [-0.23, 0.39]**

Heterogeneity: Chi² = 1.05, df = 1 (P = 0.31); I² = 5%
Test for overall effect: Z = 0.51 (P = 0.61)

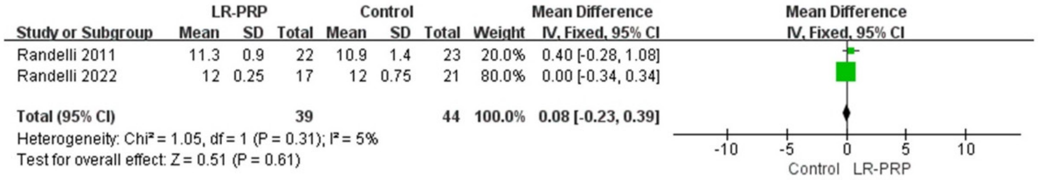

Supplementary Figure 2. Forest plots of Simple Shoulder Test.

Study or Subgroup	LR-PRP Mean	LR-PRP SD	LR-PRP Total	Control Mean	Control SD	Control Total	Weight	Mean Difference IV, Fixed, 95% CI
Randelli 2011	33.3	2.2	22	31.3	4.1	23	20.1%	2.00 [0.09, 3.91]
Randelli 2022	34	1.5	17	33	1.5	21	79.9%	1.00 [0.04, 1.96]
**Total (95% CI)**			**39**			**44**	**100.0%**	**1.20 [0.34, 2.06]**

Heterogeneity: Chi² = 0.84, df = 1 (P = 0.36); I² = 0%
Test for overall effect: Z = 2.75 (P = 0.006)

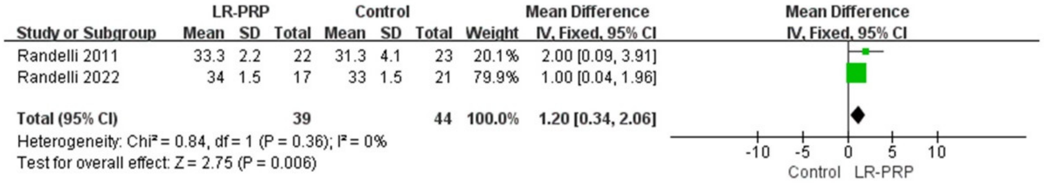

Supplementary Figure 3. Forest plots of University of California, Los Angeles score.
