# Supplementary material for: Leukocyte-Rich Platelet-Rich Plasma’s Clinical Effectiveness in Arthroscopic Rotator Cuff Repair: A Meta-Analysis of Randomized Controlled Trials
Source: Bioengineering (Basel). 2025 Jun 5;12(6):617. doi: 10.3390/bioengineering12060617 (PMC12189123; doi:10.3390/bioengineering12060617)

Supplementary material 5: The Preferred Reporting Items for Systematic reviews and Meta-analysis (PRISMA) flow diagram to show study selection.

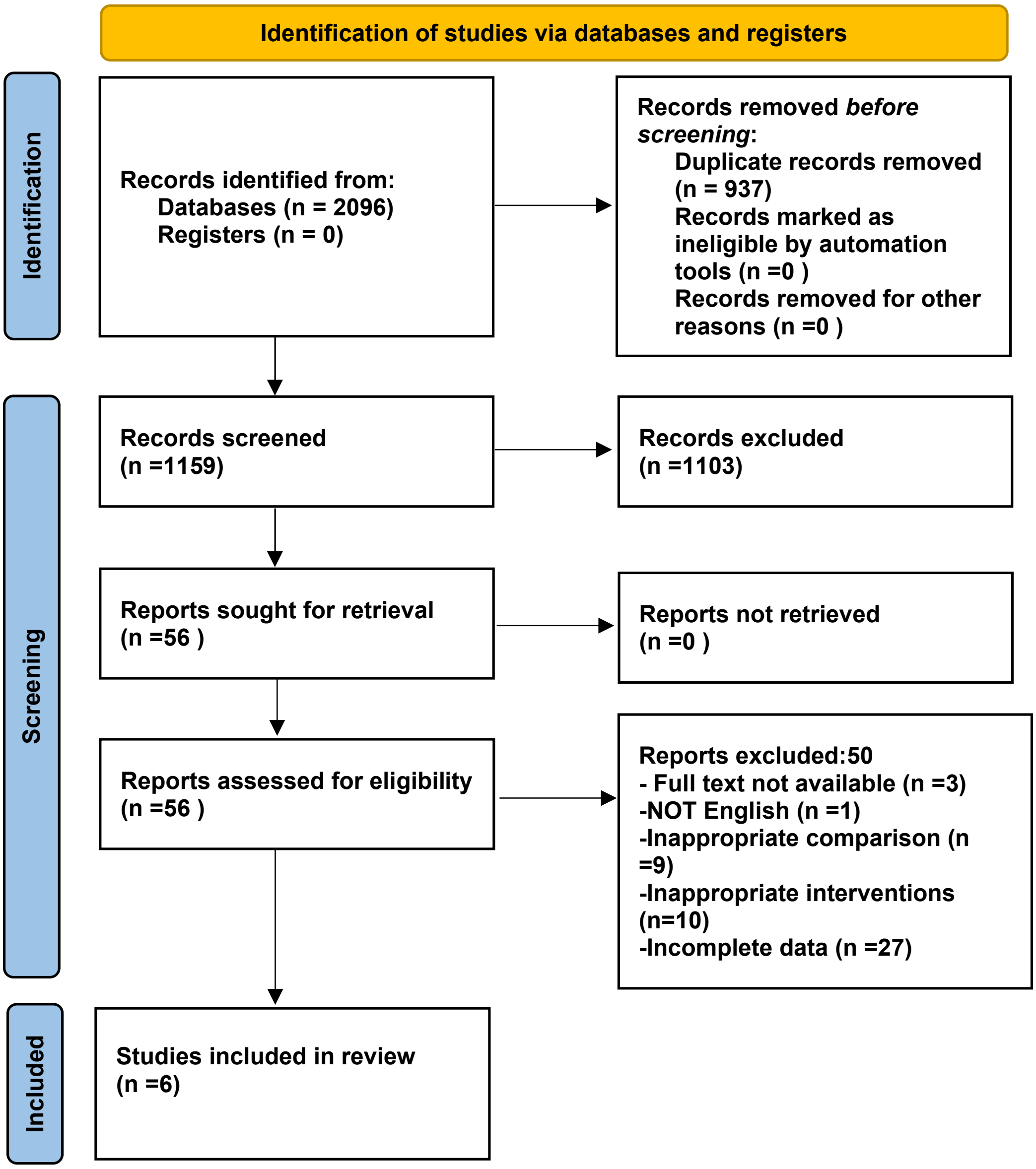

Supplement: Supplementary file 1 [file bioengineering-12-00617-s001.zip › Supplementary material 5.pdf]
